# TL1A serves as a positive regulator to promote adipocyte differentiation

**DOI:** 10.1371/journal.pone.0343036

**Published:** 2026-02-19

**Authors:** Ziqi Chang, Qiaoyu Wang, Yan Zhai, Ke Li, Xianjie Zheng, Yaohui Wang, Dan Zhao

**Affiliations:** 1 Department of Cardiovascular Surgery, The First Affiliated Hospital of Henan University, Kaifeng, China; 2 Joint National Laboratory for Antibody Drug Engineering, Henan University, Kaifeng, China; Vall d'Hebron Institut de Recerca, SPAIN

## Abstract

Adipogenesis, the intricate process of differentiation from preadipocytes or mesenchymal stem cells into mature adipocytes, is crucial for the formation and metabolic function of adipose tissues in mammals. The TNF ligand–related molecule 1A (TL1A) is a type II transmembrane protein belonging to the TNF superfamily. Inflammation is involved in the whole process of adipocyte cell formation and obesity development. To investigate the potential influence of TL1A on adipocyte development, we examined mouse embryo fibroblasts (MEFs) and 3T3-L1 cells. Our findings indicated that TL1A-treated MEFs exhibited an elevated rate of spontaneous adipogenesis, with a significant enhancement in adipocyte formation upon induction with a combination of insulin, dexamethasone and methylisobutylxanthine. This increased adipogenesis was evidenced by augmented lipid droplet formation and elevated expression of several adipogenic markers. Specifically, there was an upregulation of early-stage adipogenesis genes, including Krox20, KLF5, C/EBPβ and C/EBPδ, as well as late-stage adipogenesis regulators such as KLF15, C/EBPα, PPARγ and aP2. Moreover, TL1A significantly upregulated the protein expression of adipogenic markers (C/EBPα, C/EBPβ, PPARγ, CD36 and aP2) in MEFs and 3T3-L1 cells. Mechanistically, TL1A enhanced the phosphorylation of yes-associated protein 1 (YAP1), which led to cytoplasmic retention. Ultimately, TL1A inhibited the stabilization and nuclear transfer of β-catenin in MEFs, probably through regulating the upstream protein YAP1. Taken together, TL1A promoted adipogenic differentiation of MEFs and 3T3-L1 cells in vitro, which partly via inhibiting YAP1 mediated β-catenin signaling pathway.

## 1. Introduction

The prevalence of obesity has risen significantly and is now acknowledged as a risk factor for numerous diseases, including diabetes, cardiovascular disease and cancer [1]. This obesity epidemic has heightened interest in adipose tissue and the process of fat cell development, known as adipogenesis. Mouse embryonic fibroblasts (MEFs) and the 3T3-L1 murine preadipocyte cell line are frequently utilized models for investigating the subcellular pathways involved in the differentiation of preadipocytes, a process known as adipogenesis. A key feature of adipogenesis is the intracellular accumulation of membrane-bound lipid droplets [2].

Adipogenesis is regulated by a complex network of transcription factors and generally comprises two steps [3–5]. The initial stage is commitment, which involves the differentiation of pluripotent or multipotent stem cells into preadipocytes. The subsequent stage is terminal differentiation, wherein preadipocytes mature into adipocytes. During adipocyte differentiation, in the presence of hormonal inducers, growth arrested preadipocytes undergo mitotic clonal expansion followed by the activation of the master regulator genes peroxisome proliferator-activated receptor γ (PPARγ) and CCAAT enhancer-binding protein α (C/EBPα) [4–6]. These factors induce downstream target genes such as fatty acid binding protein 4 (FABP4/aP2), which lead to the formation of differentiated adipocytes. In response to adipocytic inducers, the early transcription factors C/EBPβ, C/EBPδ and PPARγ1 are rapidly activated or induced, initiating the adipogenic cascade. This cascade includes the increased expression of two critical transcription factors responsible for adipogenesis: C/EBPα and PPARγ2, followed by upregulation of downstream genes characteristic of mature adipocytes, including aP2, glucose transporter type 4 (Glut4) and adiponectin [5,7].

In addition to the aforementioned positive regulators, specific signaling pathways function as negative regulators of adipogenesis. For instance, the Wnt/β-catenin signaling pathway serves as a significant inhibitor of adipogenesis [8]. The inactivation of this pathway, such as through the silencing β-catenin, promotes adipogenesis in human bone marrow-derived stromal cells [9]. Conversely, the stabilization of β-catenin in murine 3T3-L1 preadipocytes inhibits their differentiation into mature adipocytes by reducing the expression of C/EBPα and PPARγ [10]. Recent studies suggest that yes-associated protein 1 (YAP1), a crucial transcriptional coactivator of the Hippo signaling pathway, plays significant roles in cell growth, differentiation and organ size control [11,12]. Generally, YAP1 can translocate from the cytoplasm to the nucleus and associate with various transcription factors [13]. When the Hippo signaling pathway is activated, the cascade of signals leads to the phosphorylation of YAP1, promoting its sequestration and degradation in the cytoplasm [14]. Overexpression of YAP1 contributes to a decrease in adipogenic differentiation. YAP1 enhances the stabilization and nuclear translocation of β-catenin, which in turn promotes osteogenic differentiation and inhibits adipogenic differentiation [15].

TNF ligand–related molecule 1A (TL1A), also referred to as vascular endothelial growth inhibitor or tumor necrosis factor superfamily 15, is classified as a type II transmembrane protein. This molecule is primarily synthesized by endothelial cells in response to inflammatory cytokines [16,17]. In addition to its role in regulating neovascularization, TL1A also promotes the formation of macrophage/foam cells in atherosclerosis [18]. TL1A is a candidate cytokine for mediating inflammatory effects in chronic inflammatory diseases such as rheumatoid arthritis and inflammatory bowel diseases [19,20]. Additionally, obesity is recognized as an inflammatory predisposition [21]. Previous studies have reported TL1A can promote the onset and progression of diet-induced obesity by regulating the local immune-inflammatory balance within adipose tissue [22]. This further confirms the critical regulatory role of TL1A in obesity and related metabolic disorders. Nevertheless, whether TL1A directly affects adipogenesis is still unclear. Therefore, in this study, we utilized MEFs and 3T3-L1 cells to investigate the effect of TL1A on adipocyte formation and to elucidate the underlying mechanisms.

## 2. Material and methods

### 2.1 Materials

Human TL1A recombinant protein (Cat# 1319-TL-010/CF) was purchased from R&D Systems. Rabbit anti-C/EBPα (Cat# A0904), C/EBPβ (Cat# A0711), β-catenin (Cat# A11932), phospho-YAP1-S127 (Cat# AP0489), YAP1 (Cat# A1002) polyclonal antibodies were purchased from ABclonal Technology (Wuhan, Hubei, China). Rabbit anti-CD36 (Cat# 18836–1-AP), Lamin A/C (Cat# 10298–1-AP), PPARγ1/2 (Cat# 16643–1-AP) polyclonal antibodies, mouse horseradish peroxidase (HRP)-conjugated glyceraldehyde-3-phosphate dehydrogenase (GAPDH, Cat# HRP-60004) monoclonal antibody and HRP-conjugated goat anti-rabbit IgG (H + L, Cat# A00001-2) antibody were purchased from Proteintech Group Inc (Chicago, IL, USA). Goat anti-adipocyte aP2 (Cat# sc-18,661) polyclonal antibody was purchased from Santa Cruz Biotechnology, Inc. (Santa Cruz, CA, USA). 3-Isobutyl-1-methylxanthine (IBMX) (Cat# 213942) and insulin (Cat# I2643) were purchased from Sigma-Aldrich. Dexamethasone (Cat# D1693) was purchased from LKT Laboratories (St. Paul, MN).

### 2.2 Preparation of primary MEFs

Primary MEFs were generated from wild-type mouse embryos at 13.5 days. Following the dissection of the head and visceral organs, the embryos were minced and digested with 0.05% trypsin/1 mM EDTA for 30 minutes at 37°C. The resulting cell suspension was centrifuged at 1000 g for 5 minutes, and the pellet was subsequently resuspended in culture medium prior to plating. The cells were plated and maintained in high glucose DMEM (Invitrogen) supplemented with 10% (v/v) fetal bovine serum (FBS) (Life Technologies) and 100 units/ml penicillin/streptomycin (Invitrogen) at 37°C in a 5%CO_2_ atmosphere. MEFs were cryopreserved in liquid nitrogen at passage 1 in aliquots of 1 × 10^6^ cells per vial.

### 2.3 Cell culture and adipogenesis induction

3T3-L1 cells, a murine fibroblast/preadipocyte cell line, were obtained from American Type Culture Collection (ATCC, Rockville, MD, USA). Passage 1 MEFs were thawed, and subsequent experiments were conducted at passage 3 or 4. Both MEFs and 3T3-L1 cells were cultured in 100 mm Petri dishes using Dulbecco’s modified Eagle’s medium (DMEM) supplemented with 10% fetal bovine serum (FBS), 50 μg/mL of penicillin/streptomycin and 2 mM glutamine. Differentiation was induced as previously described [18]. Two days post-confluence, preadipocytes (designated as day 0) were treated with DMEM containing 10% (v/v) FBS (Gibco), 10 μg/mL insulin (Biological Industries), 1 μM dexamethasone (Sigma) and 0.5 mM 3-isobutyl-1-methylxanthine (IBMX, Sigma) (designated as MDI) for a duration of 2 days. Subsequently, the medium was replaced with DMEM supplemented with 10% FBS and 1 μg/mL insulin for another 2 days. The culture medium was replenished with fresh DMEM containing 10% FBS every 2 days until cells achieved full adipocyte morphology. Both MEFs and 3T3-L1 cells were treated with different concentration of human TL1A recombinant protein (0, 10, 20, 50, 100, 200 ng/mL) for 3 days or 8 days in differentiation media, unless otherwise stated. The control samples were treated with equivalent concentrations of solvents.

### 2.4 Oil Red O staining

MEFs and 3T3-L1 preadipocytes were induced to undergo differentiation as previously described. To assess lipid accumulation, Oil Red O staining was conducted on the seventh day post-induction. The differentiated cells were fixed using 4% paraformaldehyde and subsequently stained with Oil Red O solution (Sigma-Aldrich). Excess stain was removed by a sequential washing process involving a single with 60% isopropanol followed by two washes with water. The plates were then scanned for analysis. For spectrophotometric quantification, the lipid-bound stain was dissolved in 100% isopropanol for 10 minutes, and the optical density was measured at 490 nm.

### 2.5 Determining the triglyceride (TG) content

MEFs or 3T3-L1 cells were homogenized by sonication and triglyceride content was measured using a commercial kit (Nanjing Jiancheng Bioengineering Institute, A110-1–1) according to the manufacturer’s instructions [23].

### 2.6 Western blotting

For Western blot analysis, cells were harvested following the induction of differentiation. Total cellular protein was extracted to assess the expression of adipocyte markers. Nuclear proteins from MEFs were isolated using a nuclear extraction kit (Solarbio) according to the manufacturer’s protocol. After quantifying the protein content, an equal amount from each sample was utilized for protein expression analysis via Western blotting [24]. For immunodetection, membranes were cut prior to hybridization with antibodies to allow simultaneous probing of multiple targets. Cropped blot images were displayed in the figures, and full-length membranes were provided in Supplementary File (S1 raw images in S2 File).

### 2.7 Quantitative real-time PCR (qRT-PCR)

Total RNA was extracted from MEFs or 3T3-L1 cells using TRIzol reagent. The expression of mRNA was quantified via quantitative reverse transcription polymerase chain reaction (qRT-PCR) employing a reverse transcription kit (New England Biolabs, Ipswich, MA, USA) and SYBR Green PCR master mix (Vazyme, Nanjing, China), along with the primers specified in S1 Table. Relative mRNA levels were normalized to GAPDH mRNA in the corresponding samples.

### 2.8 Data analysis

Each experiment was conducted in triplicate, and representative results were presented. The data were presented as the mean ± standard deviation (S.D.). Statistical differences were determined using Student’s t-test or one-way ANOVA. The statistical analyses were performed using GraphPad software, with a significance threshold set at *P* < 0.05. Significance levels are indicated as followed: *P < 0.05; **P < 0.01; ***P < 0.001.

## 3. Results

### 3.1 TL1A enhances adipocyte differentiation, accumulation of neutral lipids and triglycerides

**Fig 1:**
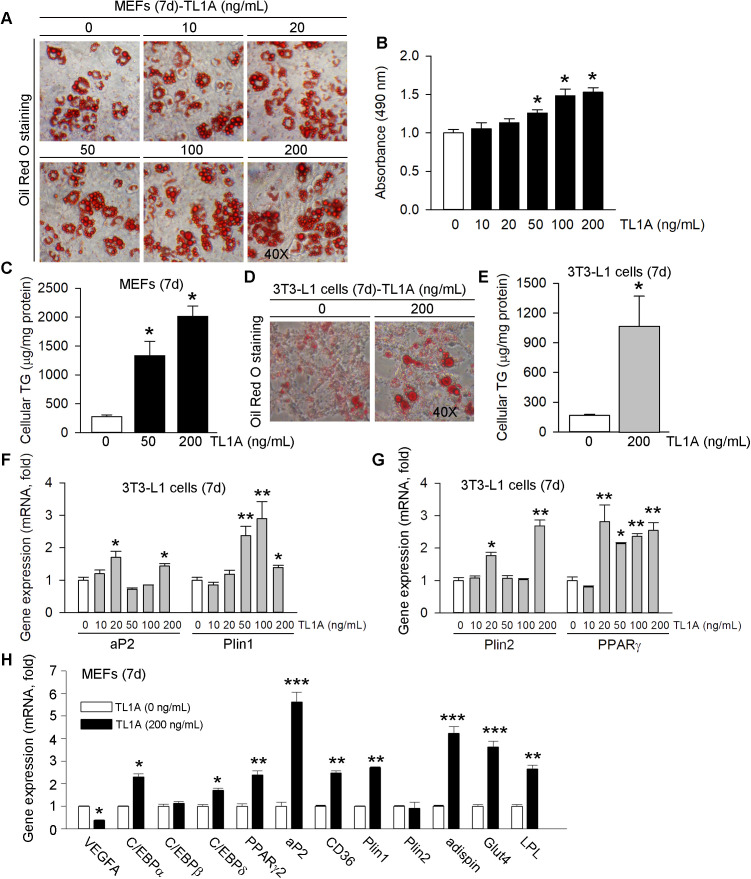
Effects of TL1A on adipogenic differentiation of MEFs and 3T3-L1 cells. Two days post-confluent MEFs or 3T3-L1 cells were cultured in adipogenic induction medium supplemented with TL1A recombinant protein at the specified concentrations (0, 10, 20, 50, 100, 200 ng/mL). (A) Oil Red O staining was conducted after adipogenic induction for 7 days. (B) The average optical density (OD) was measured using a Universal Microplate Spectrophotometer at 490 nm. (C) Measurement of triglyceride (TG) content in MEFs 7 days after adipogenic differentiation. (D-E) Both Oil Red O staining and triglyceride assays in 3T3-L1 cells were performed on day 7 of differentiation. (F-G) qRT-PCR analysis was conducted to evaluate the expression of adipogenic markers aP2, Plin1, Plin2 and PPARγ in 3T3-L1 cells 7 days after adipogenic differentiation. (H) qRT-PCR analysis was conducted to evaluate the expression of VEGFA and several adipogenic markers C/EBPα/β/δ, PPARγ2, aP2, CD36, Plin1, Plin2, adispin, Glut4 and LPL in MEFs 7 days after adipogenic differentiation. *P < 0.05, **P < 0.01, ***P < 0.001 *vs*. the group of adipocytes without TL1A treatment (n = 3).

Primary MEFs are widely utilized in studies of adipogenesis due to their multipotent nature, allowing them to differentiate into adipocytes. To investigate the effect of TL1A on adipocyte differentiation, TL1A (0, 10, 20, 50, 100, 200 ng/mL) was administered to MEFs at the onset of differentiation induced by the adipogenic cocktail (MDI). An equal volume of phosphate-buffered saline (PBS), serving as the vehicle, was added to the control group. This treatment was maintained until differentiation was fully achieved. The impact of TL1A on adipocyte differentiation was assessed through Oil Red O staining and analysis of cellular triglyceride (TG) content. Abundant oil droplets were observed in differentiated MEFs. Notably, TL1A at a concentration of 50 ng/mL significantly increased the quantity of oil droplets, while a concentration of 200 ng/mL resulted in complete induction of oil droplet formation (Figs 1A and 1B). In accordance with the observed oil droplet formation, TL1A significantly elevated cellular TG levels in a concentration-dependent manner (Fig 1C). Both Oil Red O staining and triglyceride assays confirmed that TL1A markedly enhanced the number of oil droplets and the levels of cellular TG in 3T3-L1 cells at a concentration of 200 ng/mL TL1A (Figs 1D and 1E).

At the transcriptional level, TL1A promoted the expression of adipogenic differentiation markers, including aP2 and PPARγ, as well as the mRNA expression of lipid droplet-related proteins perilipin 1 (Plin1) and Plin2 in 3T3-L1 cells (Figs 1F and 1G). Furthermore, MEFs were treated with 200 ng/mL TL1A recombinant protein, which effectively inhibited the mRNA expression of vascular endothelial growth factor A (VEGFA), indicating its normal bioactivity. As anticipated, TL1A significantly upregulated the mRNA expression of several adipocyte marker genes, such as C/EBPα, C/EBPβ, C/EBPδ, PPARγ2, aP2, CD36, Plin1, adispin, Glut4 and LPL (Fig.1H). These results thus corroborate that TL1A plays an activating role in adipogenesis.

### 3.2 Master regulators of adipogenesis are induced in TL1A-treated MEFs and 3T3-L1 cells

**Fig 2:**
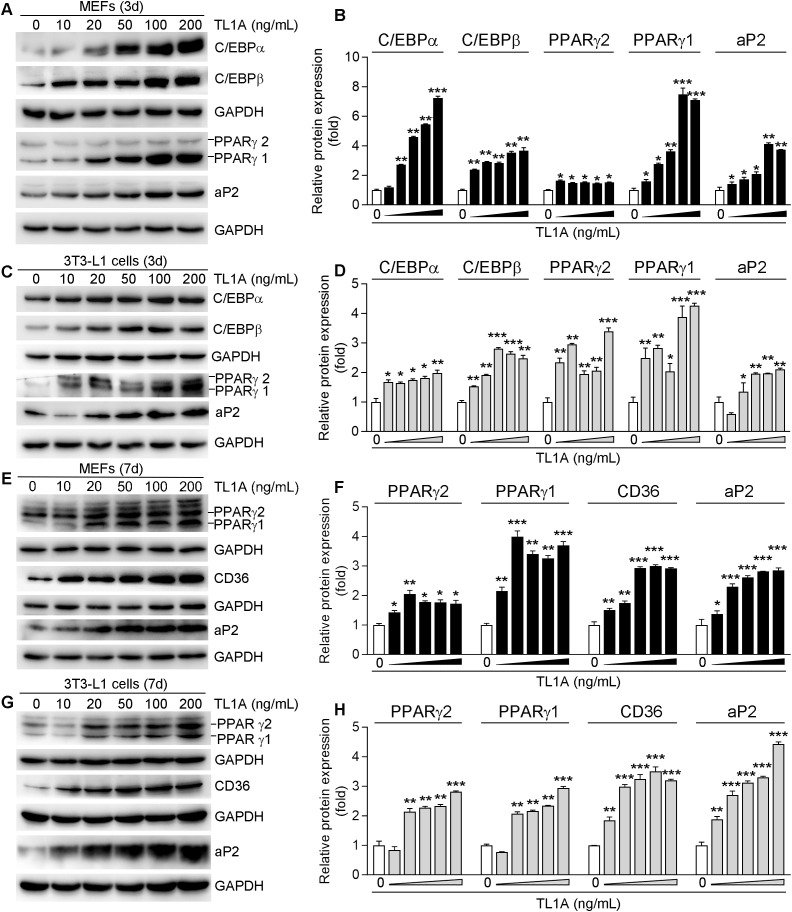
TL1A induces adipogenic markers expression during adipocyte differentiation. (A-D) MEFs or 3T3-L1 cells were induced to undergo adipocyte differentiation for 3 days in the presence of the specified concentrations of TL1A. Total protein was extracted to determine C/EBPα, C/EBPβ, PPARγ2, PPARγ1 and aP2 protein expression by Western blotting with quantitation of band density. (E-H) MEFs or 3T3-L1 cells were induced to undergo adipocyte differentiation for 7 days with the indicated concentrations of TL1A. Total protein was extracted to determine PPARγ2, PPARγ1, CD36 and aP2 protein expression by Western blottingwith quantitation of band density. *P < 0.05, **P < 0.01, ***P < 0.001 *vs*. the group of adipocytes without TL1A treatment (n = 3).

In order to investigate the effect of TL1A on white adipogenic differentiation, MEFs and 3T3-L1 cells were subjected to white adipogenesis induction, as detailed in the Methods section, for a period of 3 days. Notably, the exogenous administration resulted in a concentration-dependent increase in the protein expression of key molecules associated with white adipogenic differentiation, including C/EBPα, C/EBPβ, PPARγ1/2 and aP2, during the adipogenic induction phase (Figs 2A-D). This observation suggests a potential role for TL1A in the regulation of adipogenic differentiation. The pro-adipogenic effects of TL1A were further elucidated through the assessment of adipogenic differentiation marker expression in MEFs and 3T3-L1 cells. Specifically, the expression of PPARγ1/2, along with two PPARγ-responsive genes, CD36 and aP2, was analyzed via Western blotting. After 7 days of induction, the protein levels of PPARγ1/2, aP2 and CD36 were significantly elevated in the presence of TL1A, reaching their peak expression at this time point (Figs 2E-H). Collectively, these findings indicate that TL1A enhances the expression of genes involved in adipogenesis.

Interestingly, our findings also demonstrated that TL1A significantly upregulated the expression of ATP-Binding Cassette A1 transporter (ABCA1) and ATP-Binding Cassette G1 transporter (ABCG1) in MEFs, indicating that TL1A may enhance lipid transport and storage capacity, thereby providing the material basis for lipid droplet formation and TG accumulation (S1 Fig).

### 3.3 Both early and late stage adipogenesis regulators are hyperinducible in TL1A-treated MEFs

**Fig 3:**
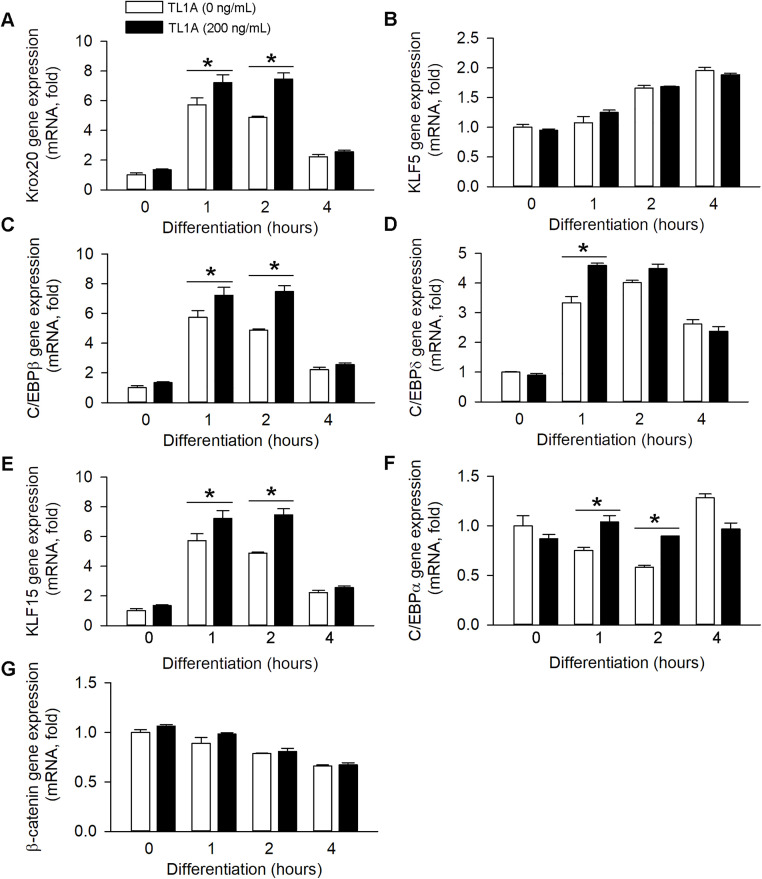
qRT-PCR analysis of adipogenesis regulators during differentiation induction. After two days post-confluence, MEFs were treated with standard differentiation medium and TL1A recombinant protein (0, 10, 20, 50, 100, 200 ng/mL). At specified time points, cells were harvested for RNA isolation and subsequent qRT-PCR analysis. *P < 0.05 *vs*. the group of adipocytes without TL1A treatment.

To elucidate the mechanism underlying the enhanced adipogenesis observed in TL1A-treated cells, we assessed the expression of genes associated with the adipogenesis cascade following induction with the MDI mixture, utilizing qRT-PCR. Consistent with our hypothesis, TL1A treatment resulted in increased mRNA expression of early adipogenic marker genes, including early growth response 2 (Krox20), Krüppel-like factor 5 (KLF5), C/EBPβ and C/EBPδ (Figs 3A-D). A similar expression pattern was observed for two late-stages of adipogenic marker genes, Krüppel-like factor 15 (KLF15) and C/EBPα (Figs 3E and 3F). Previous studies have demonstrated that β-catenin inhibits the expression of anti-adipogenic genes [25]. Consequently, we investigated whether reduced β-catenin gene expression could explain the enhanced adipogenesis in TL1A-treated MEFs. Unexpectedly, TL1A treatment did not affect β-catenin mRNA expression during adipocyte differentiation (Fig 3G). Collectively, these findings suggest that TL1A augments the expression of several factors involved in both the early and late stages of adipocyte differentiation.

### 3.4 TL1A enhances adipogenesis by inhibiting YAP1 signaling

**Fig 4:**
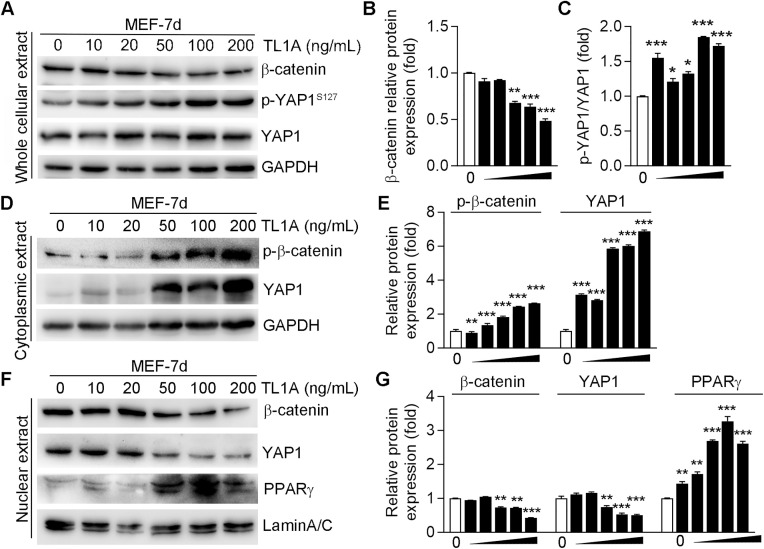
TL1A inhibits β-catenin expression by promoting the phosphorylation of YAP1. MEFs were treated with standard differentiation medium and TL1A at the specified concentrations (0, 10, 20, 50, 100, 200 ng/mL) for 7 days. (A) Total cellular proteins were extracted, and the expression levels of β-catenin, phosphorylated YAP1^S127^ (p-YAP1^S127^) and total YAP1 proteins were determined by Western blotting. (B) The relative amounts of β-catenin protein expression were calculated according to the grayscale values and were showed in the histogram. (C) The quantitation of ratio (p-YAP1^S127^/total YAP1) to reflect the inactivation of YAP1 signaling pathway. (D-E) At the end of adipogenesis induction, protein expression of phosphorylated β-catenin (p- β-catenin) and YAP1 in the cytoplasm extract from MEFs was determined by Western blotting. (F-G) Nuclear extracts were isolated from the MEFs and analyzed for protein expression by Western blotting. Nuclear proteins were isolated to evaluated the expression of β-catenin, YAP1 and PPARγ proteins through Western blotting, accompanied by a quantitative analysis of band density. GAPDH and Lamin A/C served as the internal controls for cytoplasmic and nuclear extract, respectively. All the histograms represent the relative expression levels of proteins normalized by GAPDH or Lamin A/C. *P<0.05, **P<0.01, ***P<0.001 *vs*. the group of MEFs without TL1A treatment.

Several studies reported that YAP1 inhibits adipogenic differentiation by modulating β-catenin signaling [26]. In comparison to control cells, TL1A-treated MEFs exhibited a marked decrease in β-catenin protein levels and an increase in phosphorylated YAP1 at serine 127 (p-YAP1^S127^). Furthermore, the p-YAP1/YAP1 ratio in MEFs increased after TL1A stimulation, though total YAP1 expression was slightly elevated, indicating that YAP1 was inactivated (Figs 4A-C). Next, we validated the expression and translocation status of β-catenin and YAP1 in MEFs. The results demonstrated that higher expression of p-β-catenin and YAP1 in the cytoplasm in TL1A-treated MEFs than in MEFs in control group (Figs 4D-E). Furthermore, TL1A significantly decreased nuclear β-catenin and YAP1 expression, but increased expression of PPARγ (Figs 4C and 4D), indicating the potential role of YAP1-β-catenin signaling in the suppression of adipogenic differentiation. These findings suggest that TL1A effectively inactivates YAP1 signaling and facilitates the phosphorylation and degradation of cytoplasmic β-catenin.

## 4. Discussion

**Fig 5:**
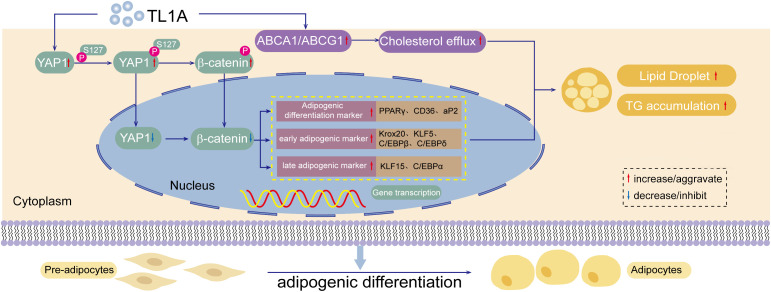
TL1A serves as a key positive regulator of adipocyte differentiation. Exogenous TL1A triggers the phosphorylation of YAP1 at Ser127, resulting in its cytoplasmic retention and functional inactivation. This event subsequently reduces β-catenin stability and prevents its nuclear translocation, thereby triggering the expression of essential adipogenic regulators and lipid metabolism-related proteins. Additionally, TL1A-mediated regulation of ABCA1 and ABCG1 expression facilitates cholesterol homeostasis, providing essential substrates for lipid droplet biogenesis. Collectively, these molecular mechanisms orchestrate the differentiation of MEFs and 3T3-L1 preadipocytes into terminally differentiated adipocytes.

This study systematically examined the role and preliminary mechanisms of TL1A in adipocyte differentiation, also known as adipogenesis. Through in vitro cellular experiments utilizing MEFs and 3T3-L1 preadipocytes, the study confirmed the promotive effect of TL1A on adipogenesis at phenotypic, molecular and signaling pathway levels. This research identifies a novel molecular target for elucidating the regulatory network of adipogenesis. The multi-dimensional experimental approach demonstrated that TL1A serves as a key positive regulator of adipogenesis. At the cellular phenotypic level, exogenous TL1A supplementation resulted in a concentration-dependent increase in the formation of neutral lipid droplets and triglyceride accumulation in both MEFs and 3T3-L1 cells. At the molecular level, TL1A significantly upregulated the transcription and protein expression of core adipogenic differentiation markers, including PPARγ, the C/EBP family and aP2, as well as lipid droplet-associated proteins such as Plin1 and Plin2. Mechanistically, TL1A synergistically enhanced adipogenesis by inhibiting the activity of the YAP1 signaling pathway while inducing the expression of cholesterol transporters ABCA1 and ABCG1 (Fig 5). These findings are corroborated across the three levels of analysis.

TL1A (also known as tumor necrosis factor superfamily member 15 [TNFSF15]) is a member of the TNF ligand superfamily and, like other members, is one of the pro-inflammatory cytokines [27]. As expected, TL1A significantly induced the expression of pro-inflammatory cytokines, such as TNFα, IL-1β and IL-6, in the MEFs and 3T3-L1 cells during adipogenic differentiation (S2 Fig). So, we found that regulation of inflammatory response and TL1A may play a vital role in the adipogenic differentiation. In addition, the primary phenotypic characteristics of adipogenesis include the formation of lipid droplets and the accumulation of triglycerides (TG) [28–30]. Our findings corroborated the regulatory influence of TL1A, as demonstrated through Oil Red O staining and TG content analysis. In MEFs, a concentration of 50 ng/mL TL1A markedly increased the number of lipid droplets, while 200 ng/mL TL1A fully induced lipid droplet formation. Concurrently, TL1A elevated intracellular TG levels in a concentration-dependent manner, an effect also observed in 3T3-L1 cells. At the molecular level, TL1A upregulated the mRNA expression of adipogenic markers such as aP2 and PPARγ, as well as proteins associated with lipid droplets, including Plin1 and Plin2, thereby reinforcing its role in promoting adipogenesis (Fig 1).

A potential limitation of this study is the concentration of recombinant TL1A (0–200 ng/mL) utilized in our in vitro models. In healthy individuals, the median serum concentration of TL1A is reported to be approximately 316.9 pg/mL. However, in inflammatory conditions such as rheumatoid arthritis, systemic levels can exceed 1 ng/mL [31]. It is important to acknowledge that obesity is now recognized as a chronic low-grade inflammatory disorder [32]. Within the adipose tissue microenvironment, the local concentration of TL1A—secreted by infiltrating immune cells—is likely significantly higher than the diluted levels observed in systemic circulation. The selection of the ng/mL gradient in our experiments was designed to simulate these pathological microenvironments. Additionally, higher doses in cell culture are often required to elicit measurable biological responses within a limited experimental timeframe, effectively modeling the long-term cumulative impact of chronic inflammation in vivo. Consequently, our findings underscore the potential role of TL1A in driving adipose metabolic dysfunction under pathological conditions, rather than maintaining healthy physiological homeostasis.

The process of adipogenesis is meticulously regulated by key transcription factors, including PPARγ and the C/EBP family (C/EBPα, C/EBPβ and C/EBPδ), with alterations in their expression levels directly influencing adipogenic efficiency [33]. In this study, it was observed that during the differentiation of white adipocytes from MEFs and 3T3-L1 cells, TL1A upregulated the protein levels of C/EBPα, C/EBPβ, PPARγ1/2 and aP2 in both a concentration-dependent and time-dependent manner. Specifically, as the concentration of TL1A increased, the expression of these proteins was progressively enhanced. Furthermore, as the differentiation period extended up to 7 days, the protein expression of PPARγ1/2, aP2 and the PPARγ target gene CD36 reached their peak levels, with significantly higher expression in the TL1A-treated group compared to the control group (Fig 2). These findings suggest that TL1A may facilitate the conversion of preadipocytes into mature adipocytes by activating the core regulatory network governing adipogenesis.

Adipogenesis is a sequential process comprising early initiation, intermediate regulation and late maturation phases, with the expression of specific markers at each stage indicating the progression of differentiation [34]. Our investigation revealed that TL1A markedly upregulated the mRNA expression of both early adipogenic marker genes (Krox20, KLF5, C/EBPβ, C/EBPδ) and late adipogenic marker genes (KLF15, C/EBPα) (Fig 3). These findings suggest that TL1A may play a role in both the initiation and maturation phases of adipogenesis, rather than being restricted to a single phase. Furthermore, prior research has established that β-catenin inhibits adipogenesis by repressing the expression of pro-adipogenic genes [35]. However, our study found that TL1A did not significantly affect the mRNA expression of β-catenin, thereby excluding the mechanism whereby “TL1A promotes adipogenesis by downregulating β-catenin transcription.” This result offers a new direction for future investigations into alternative pathways.

To elucidate the molecular mechanisms by which TL1A modulates adipogenesis, this study concentrated on lipid transport-associated proteins and signaling pathways. Notably, the membrane protein ABCA1 serves as the principal pathway for cholesterol efflux in adipocytes, whereas the membrane protein ABCG1 influences adipogenesis by facilitating LPL-dependent triglyceride (TG) storage and activating the PPARγ-mediated adipogenic transcriptional program [36,37]. While our data demonstrate that TL1A regulates the expression of ABCA1 and ABCG1 (S1 Fig), further studies are required to elucidate their direct functional roles in lipid accumulation during TL1A-mediated adipocyte differentiation. Conversely, the YAP1 signaling pathway functions as a critical negative regulator of adipogenesis, with its activation promoting osteogenesis and inhibiting adipogenesis [38]. This study observed that TL1A markedly increased the phosphorylation level of YAP1 at serine 127 and facilitated the nuclear localization of β-catenin and PPARγ, while only slightly elevating the total protein level of YAP1 in MEFs. In conjunction with the findings presented in Fig. 3G, which showed that TL1A did not affect β-catenin mRNA levels, these results suggest that TL1A may regulate β-catenin through post-translational modifications. Taken together, TL1A causes the phosphorylation of YAP1, then promotes the sequestration and degradation of YAP1 in cytoplasm, thus the inactivation of the YAP1 facilitates the phosphorylation and degradation of cytoplasmic β-catenin.

Based on the preliminary mechanistic findings of this study, future research will focus on a comprehensive exploration of the regulatory mechanisms of the YAP1-β-catenin pathway to elucidate the molecular details of TL1A-regulated adipogenesis. Subsequent in vivo experiments, including the analysis of adipose tissue development in TL1A knockout mice and the investigation of adipogenesis in obesity models, are necessary to validate the physiological function of TL1A, thereby providing a theoretical foundation for understanding the pathogenesis of lipid metabolism-related diseases.

## 5. Conclusion

This study represents the inaugural confirmation that TL1A augments adipogenesis via inactivation of the YAP1 signaling pathway and facilitates the phosphorylation and degradation of cytoplasmic β-catenin. Collectively, these findings substantiate the promotive role of TL1A in adipogenesis via exogenous supplementation. Consequently, TL1A may hold potential as a candidate for drug design and clinical applications targeting obesity-related diabetes, hyperlipidemia and other metabolic syndromes.

## Supporting information

S1 Fig TL1A increases the expression of cholesterol efflux-related markers. Two days post-confluence, MEFs were treated for 7 days with an adipogenic cocktail (MDI), including 10 μg/mL insulin (Biological Industries), 1 μM dexamethasone (Sigma) and 0.5 mM 3-isobutyl-1-methylxanthine (IBMX, Sigma) with or without TL1A treatment at the indicated concentrations. After treatment, expression of ATP-binding cassette transporter A1 (ABCA1) and ABCG1 was determined by Western blotting (A) with quantitation of band density (B). *P < 0.05, **P < 0.01, ***P < 0.001 *vs*. the group of adipocytes without TL1A treatment (n = 3).
(PDF)

S2 Fig TL1A promotes the expression of inflammatory factors in both MEFs and 3T3-L1 cells. Two days post-confluence, MEFs and 3T3-L1 cells were treated for 7 days with an adipogenic cocktail (MDI), including 10 μg/mL insulin (Biological Industries), 1 μM dexamethasone (Sigma) and 0.5 mM 3-isobutyl-1-methylxanthine (IBMX, Sigma) with or without TL1A treatment at 200 ng/mL. (A-B) qRT-PCR analysis of the indicated cytokines (TNFα, IL-1β and IL-6) after adipogenic induction for 7 days. ***P < 0.001 *vs*. the group of adipocytes without TL1A treatment (n = 3).
(PDF)

S1 Table Sequences of the primers for qRT-PCR analysis. m, mus musculus; FABP4/aP2, Fatty acid binding protein 4; C/EBPα, CCAAT/enhancer-binding protein α; C/EBPβ, CCAAT/enhancer-binding protein β; C/EBPδ, CCAAT/enhancer-binding protein δ; Glut4, Glucose transporter type 4; KLF5, Krüppel-like factor 5; KLF15, Krüppel-like factor 15; Krox20, Early growth response 2; LPL, Lipoprotein lipase; Plin 1/2, Perilipin 1/2; PPARγ1/2, peroxisome proliferator-activated receptorγ1/2; VEGFA, vascular endothelial growth factor A; TNFα, tumor necrosis factor alpha; IL-1β, interleukin 1 beta; IL-6, interleukin 6; GAPDH, glyceraldehyde-3-phosphate dehydrogenase.
(PDF)

S1 File Supplemental experimental materials. Rabbit anti- ABCA1 (Cat# A16337) polyclonal antibody was purchased from ABclonal Technology (Wuhan, Hubei, China). Rabbit anti- ABCG1 (CAT# 13578–1-AP) polyclonal antibody was purchased from Proteintech Group Inc (Chicago, IL, USA).
(PDF)

S2 Flie S1 raw images. Membranes were cut prior to hybridization with antibodies to allow simultaneous probing of multiple targets. Cropped blot images of the original western blots were displayed in the figures, and full-length membranes were provided.
(PDF)
