# Supplementary material for: TL1A serves as a positive regulator to promote adipocyte differentiation
Source: PLoS One. 2026 Feb 19;21(2):e0343036. doi: 10.1371/journal.pone.0343036 (PMC12919779; doi:10.1371/journal.pone.0343036)
Supplement: S1 Fig — Two days post-confluence, MEFs were treated for 7 days with an adipogenic cocktail (MDI), including 10 μg/mL insulin (Biological Industries), 1 μM dexamethasone (Sigma) and 0.5 mM 3-isobutyl-1-methylxanthine (IBMX, Sigma) with or without TL1A treatment at the indicated concentrations. After treatment, expression of ATP-binding cassette transporter A1 (ABCA1) and ABCG1 was determined by Western blotting (A) with quantitation of band density (B). *P < 0.05, **P < 0.01, ***P < 0.001 vs. the group of adipocytes without TL1A treatment (n = 3). (PDF) [file pone.0343036.s001.pdf]

S1 Fig. TL1A increases the expression of cholesterol efflux-related markers. Two days post-confluence, MEFs were treated for 7 days with an adipogenic cocktail (MDI), including 10 μg/mL insulin (Biological Industries), 1 μM dexamethasone (Sigma) and 0.5 mM 3-isobutyl-1-methylxanthine (IBMX, Sigma) with or without TL1A treatment at the indicated concentrations. After treatment, expression of ATP-binding cassette transporter A1 (ABCA1) and ABCG1 was determined by Western blotting (A) with quantitation of band density (B). *P<0.05, **P<0.01, ***P<0.001 vs. the group of adipocytes without TL1A treatment (n=3).
