# Supplementary material for: TL1A serves as a positive regulator to promote adipocyte differentiation
Source: PLoS One. 2026 Feb 19;21(2):e0343036. doi: 10.1371/journal.pone.0343036 (PMC12919779; doi:10.1371/journal.pone.0343036)
Supplement: S2 Fig — Two days post-confluence, MEFs and 3T3-L1 cells were treated for 7 days with an adipogenic cocktail (MDI), including 10 μg/mL insulin (Biological Industries), 1 μM dexamethasone (Sigma) and 0.5 mM 3-isobutyl-1-methylxanthine (IBMX, Sigma) with or without TL1A treatment at 200 ng/mL. (A-B) qRT-PCR analysis of the indicated cytokines (TNFα, IL-1β and IL-6) after adipogenic induction for 7 days. ***P < 0.001 vs. the group of adipocytes without TL1A treatment (n = 3). (PDF) [file pone.0343036.s002.pdf]

S2 Fig. TL1A promotes the expression of inflammatory factors in both MEFs and 3T3-L1 cells. Two days post-confluence, MEFs and 3T3-L1 cells were treated for 7 days with an adipogenic cocktail (MDI), including 10 μg/mL insulin (Biological Industries), 1 μM dexamethasone (Sigma) and 0.5 mM 3-isobutyl-1-methylxanthine (IBMX, Sigma) with or without TL1A treatment at 200 ng/mL. (A-B) qRT-PCR analysis of the indicated cytokines (TNFα, IL-1β and IL-6) after adipogenic induction for 7 days. ***P<0.001 vs. the group of adipocytes without TL1A treatment (n=3).
