# Supplementary material for: TL1A serves as a positive regulator to promote adipocyte differentiation
Source: PLoS One. 2026 Feb 19;21(2):e0343036. doi: 10.1371/journal.pone.0343036 (PMC12919779; doi:10.1371/journal.pone.0343036)
Supplement: S1 Table — m, mus musculus; FABP4/aP2, Fatty acid binding protein 4; C/EBPα, CCAAT/enhancer-binding protein α; C/EBPβ, CCAAT/enhancer-binding protein β; C/EBPδ, CCAAT/enhancer-binding protein δ; Glut4, Glucose transporter type 4; KLF5, Krüppel-like factor 5; KLF15, Krüppel-like factor 15; Krox20, Early growth response 2; LPL, Lipoprotein lipase; Plin 1/2, Perilipin 1/2; PPARγ1/2, peroxisome proliferator-activated receptorγ1/2; VEGFA, vascular endothelial growth factor A; TNFα, tumor necrosis factor alpha; IL-1β, interleukin 1 beta; IL-6, interleukin 6; GAPDH, glyceraldehyde-3-phosphate dehydrogenase. (PDF) [file pone.0343036.s003.pdf]

S1 Table. Sequences of the primers for qRT-PCR analysis.

Genes	Primers	Sequence (5'-3')
m-adipsin	forward	GCTATCCCAGAATGCCTCGTT
	reverse	GGTTCCACTTCTTTGTCCTCGTAT
m-aP2 (FABP4)	forward	AAGGTGAAGAGCATCATAACCCT
	reverse	TCACGCCTTTCATAACACATTCC
m-β-catenin	forward	TGCCACCACCACAGCTCCTT
	reverse	GGAACATGGCAGCTCGGACCC
m-CD36	forward	CTATTGGCCAAGCTATTGCG
	reverse	TCAGATCCGAACACAGCGTA
m-C/EBPα	forward	CAAGAACAGCAACGAGTACCG
	reverse	GTCACTGGTCAACTCCAGCAC
m-C/EBPβ	forward	GGCGCGAGCGCAACAACATC
	reverse	GCTCGGGCAGCTGCTTGAACAA
m-C/EBPδ	forward	CGACTTCAGCGCCTACATTGA
	reverse	CTAGCGACAGACCCCACAC
m-Glut4	forward	GGCTGTGCCATCTTGATGAC
	reverse	AAGACGTAAGGACCCATAGCAT
m-KLF5	forward	CCGGAGACGATCTGAAACACG
	reverse	GTTGATGCTGTAAGGTATGCCT
m-KLF15	forward	CCATTGCCGCCAAACCTATTG
	reverse	AACTCATCTGAGCGGGAAAAC
m-Krox20	forward	GCCAAGGCCGTAGACAAAATC
	reverse	CCACTCCGTTCATCTGGTCA
m-LPL	forward	AGGACCCCTGAAGACAC
	reverse	GGCACCCAACTCTCATA
m-Plin1	forward	CAAGCACCTCTGACAAGGTTC
	reverse	GTTGGCGGCATATTCTGCTG
m-Plin2	forward	CTTGTGTCCTCCGCTTATGTC
	reverse	GCAGAGGTCACGGTCTTCAC
m-PPARγ1	forward	AAAGAAGCGGTGAACCACTGATA
	reverse	AATGGCATCTCTGTGTCAACCA
m-PPARγ2	forward	CGCTGATGCACTGCCTATGAG
	reverse	TGGGTCAGCTCTTGTGAATGGAA
m-VEGFA	forward	TCAGAGCGGAGAAAGCATTTGT
	reverse	GGTGACATGGTTAATCGGTCTT
m-TNFα	forward	AAAGAAGCGGTGAACCACTGATA
	reverse	AATGGCATCTCTGTGTCAACCA
m-IL-β	forward	CGCTGATGCACTGCCTATGAG
	reverse	TGGGTCAGCTCTTGTGAATGGAA
m-IL-6	forward	TCAGAGCGGAGAAAGCATTTGT

	reverse	GGTGACATGGTTAATCGGTCTT
m-GAPDH	forward	TGTGTCCGTCGTGGATCTGA
	reverse	TTGCTGTTGAAGTCGCAGGAG

m, mus musculus; FABP4/aP2, Fatty acid binding protein 4; C/EBPα, CCAAT/enhancer-binding protein α; C/EBPβ, CCAAT/enhancer-binding protein β; C/EBPδ, CCAAT/enhancer-binding protein δ; Glut4, Glucose transporter type 4; KLF5, Krüppel-like factor 5; KLF15, Krüppel-like factor 15; Krox20, Early growth response 2; LPL, Lipoprotein lipase; Plin 1/2, Perilipin 1/2; PPARγ1/2, peroxisome proliferator-activated receptor γ 1/2; VEGFA, vascular endothelial growth factor A; TNFα, tumor necrosis factor alpha; IL-1β, interleukin 1 beta; IL-6, interleukin 6; GAPDH, glyceraldehyde-3-phosphate dehydrogenase.
