# Supplementary material for: TL1A serves as a positive regulator to promote adipocyte differentiation
Source: PLoS One. 2026 Feb 19;21(2):e0343036. doi: 10.1371/journal.pone.0343036 (PMC12919779; doi:10.1371/journal.pone.0343036)
Supplement: S1 File — Rabbit anti- ABCA1 (Cat# A16337) polyclonal antibody was purchased from ABclonal Technology (Wuhan, Hubei, China). Rabbit anti- ABCG1 (CAT# 13578–1-AP) polyclonal antibody was purchased from Proteintech Group Inc (Chicago, IL, USA). (PDF) [file pone.0343036.s004.pdf]

Supplemental experimental materials

Rabbit anti- ABCA1 (Cat# A16337) polyclonal antibody was purchased from ABclonal Technology (Wuhan, Hubei, China). Rabbit anti- ABCG1 (CAT# 13578-1-AP) polyclonal antibodies and mouse horseradish peroxidase (HRP)-conjugated glyceraldehyde-3-phosphate dehydrogenase (GAPDH, Cat# HRP-60004) monoclonal antibody were purchased from Proteintech Group Inc (Chicago, IL, USA).
