# Supplementary material for: TL1A serves as a positive regulator to promote adipocyte differentiation
Source: PLoS One. 2026 Feb 19;21(2):e0343036. doi: 10.1371/journal.pone.0343036 (PMC12919779; doi:10.1371/journal.pone.0343036)

Supplementary figures (the whole uncropped images of the original western blots)

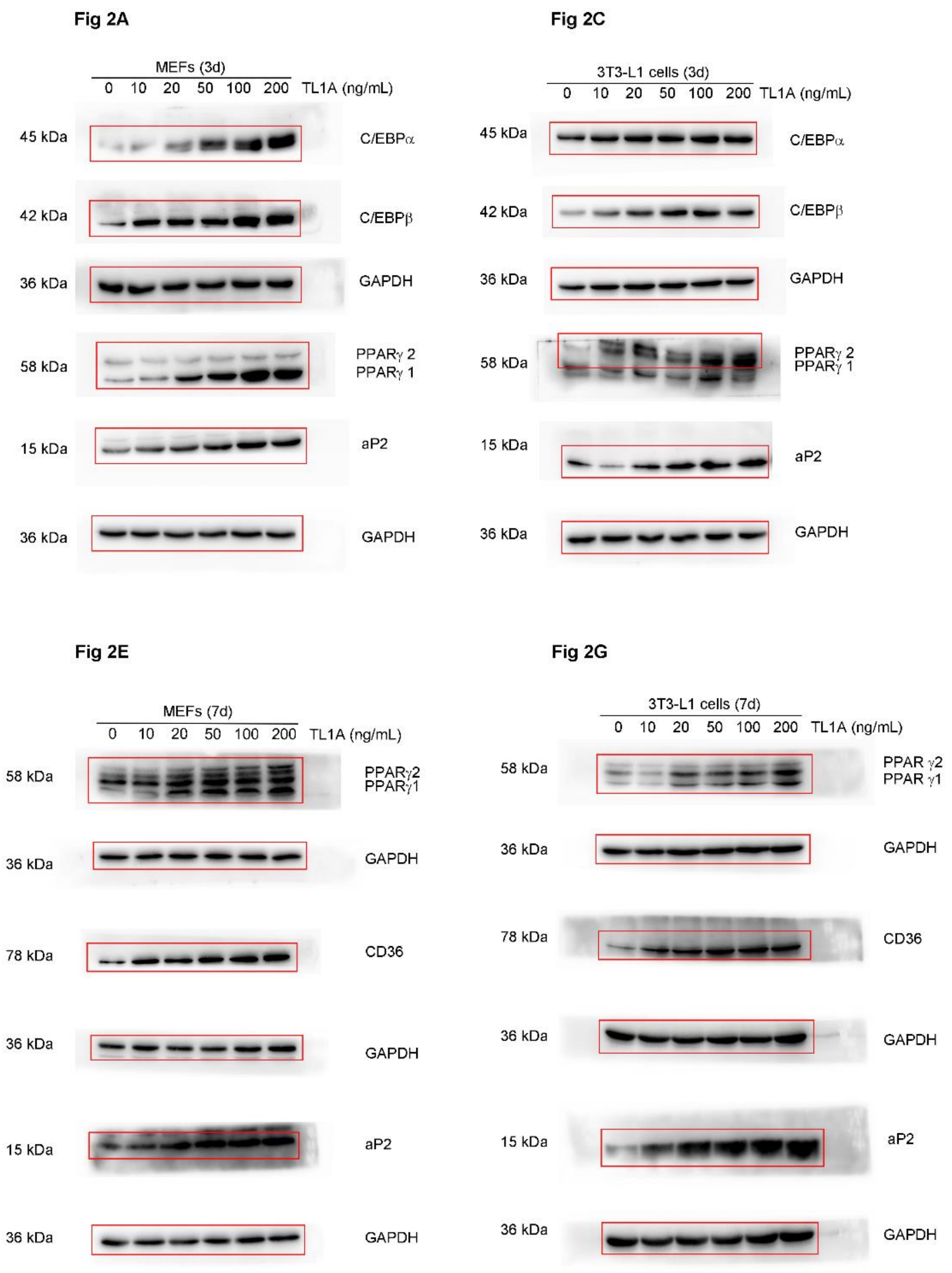

Fig 4A

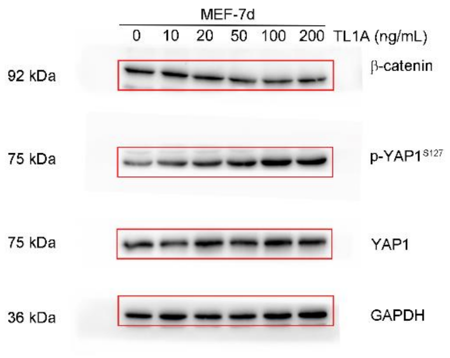

S1 Fig

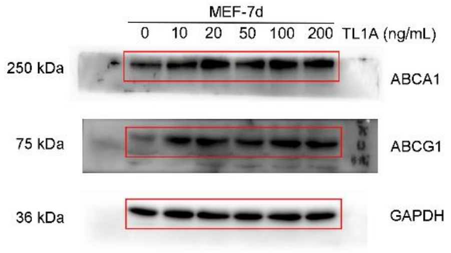

Fig 4D

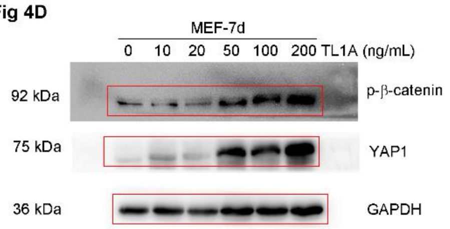

Fig 4F

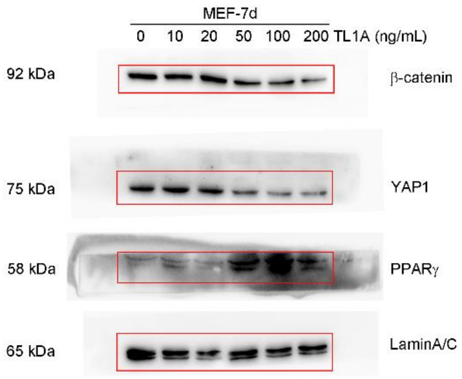

Supplement: S2 Flie — Membranes were cut prior to hybridization with antibodies to allow simultaneous probing of multiple targets. Cropped blot images of the original western blots were displayed in the figures, and full-length membranes were provided. (PDF) [file pone.0343036.s005.pdf]
